# The forgotten appearance of metastatic melanoma in the small bowel

**DOI:** 10.1186/s40644-022-00463-5

**Published:** 2022-06-14

**Authors:** Eva Mendes Serrao, Emily Joslin, Victoria McMorran, Caroline Hough, Cheryl Palmer, Sarah McDonald, Emma Cargill, Ashley S. Shaw, Brent O’Carrigan, Christine A. Parkinson, Pippa G. Corrie, Timothy J. Sadler

**Affiliations:** 1 Department of Radiology, Addenbrooke’s Hospital, Cambridge University Hospitals NHS Foundation Trust, Cambridge, UK; 2 Department of Radiology, University of Cambridge, Box 218, Cambridge, CB2 0QQ UK; 3 Department of Pathology, Addenbrooke’s Hospital, Cambridge University Hospitals NHS Foundation Trust, Cambridge, UK; 4 Department of Oncology, Addenbrooke’s Hospital, Cambridge University Hospitals NHS Foundation Trust, Cambridge, UK; 5 Department of Oncology, Hinchingbrooke Hospital, Huntingdon, UK

**Keywords:** Melanoma, Metastatic, Aneurysmal, Small bowel, Radiology

## Abstract

**Background:**

Melanoma is the most aggressive form of skin cancer, with a tendency to metastasise to any organ of the human body. While the most common body organs affected include liver, lungs, brain and soft tissues, spread to the gastrointestinal tract is not uncommon. In the bowel, it can present with a multitude of imaging appearances, more rarely as an aneurysmal dilatation. This appearance is classically associated with lymphoma, but it has more rarely been associated with other forms of malignancy.

**Case presentation:**

We report a case series of three patients with aneurysmal dilatation in the small bowel (SB) confirmed to be due to metastatic melanoma (MM). All patients had non-specific symptoms; most times being attributed initially to causes other than melanoma. On CT the identified aneurysmal SB dilatations were diagnosed as presumed lymphoma in all cases. In two cases, the aneurysmal dilatation was the first presentation of metastatic disease and in two of the cases more than one site of the gastrointestinal tract was concomitantly involved. All patients underwent surgical resection with histological confirmation of MM.

**Conclusions:**

Recognition of unusual SB presentation of MM, such as aneurysmal SB dilatation, is important to expedite diagnosis, provide appropriate treatment, and consequently improve quality of life and likely survival of these patients. As the most common cancer to metastasise to the SB and as a known imaging mimicker, MM should remain in any radiologist’s differential diagnosis for SB lesions with aneurysmal dilatation.

**Supplementary Information:**

The online version contains supplementary material available at 10.1186/s40644-022-00463-5.

## Background

Different small bowel (SB) imaging patterns have been associated with metastatic melanoma (MM). More frequently, it presents with polypoid nodules [1] causing intussusception, and less frequently as ulcerating mural nodules, exo-enteric lesions, infiltrating masses or serosal deposits. Aneurysmal dilatation is a rare presentation with only a single case previously reported in the literature to the best of our knowledge [2]. This appearance is defined as a cavitary dilatation of the intestinal lumen (> 4 cm) with a nodular, irregular luminal contour and peripheral bowel wall thickening [3] and was first described as an imaging finding characteristic of lymphoma [4]. However, it has subsequently been associated with other cancer types, including primary SB adenocarcinoma, leiomyosarcoma [5], gastrointestinal stromal tumours [6], and metastatic disease from non-small cell bronchogenic carcinoma [7], adenocarcinoma of the rectum and endometrial stromal sarcoma [8], as well as with amyloidosis [9].

Melanoma is the most aggressive form of skin cancer, with a tendency to metastasise to all organs of the human body. It is the most common metastatic tumour to the SB [10, 11]. Inevitably, most SB lesions are initially detected by CT, as the mainstream technique for staging and follow-up of melanoma patients. However, their imaging diagnosis is often challenging given the multiplicity and multitude of appearances of these lesions. Metastases to the SB, particularly in MM, can be clinically silent and can occur years after initial diagnosis and treatment [11]. However, detection of SB metastatic disease is important as patients may become symptomatic with SB obstruction, intussusception, perforation or gastrointestinal (GI) bleeding, and in some cases lead to emergency surgical intervention [12]. Additionally, early diagnosis and treatment of these lesions has been shown to improve survival and quality of life, even in palliative cases [13].

Correct and timely recognition of the different imaging patterns of SB MM can optimise and expedite patient care by guiding diagnosis, treatment and prognosis. Early diagnosis of SB metastases whilst small in size, can facilitate elective treatment and avoid non-elective hospitalisations. Here, we report the radiological and pathological findings of a series of patients found to have aneurysmal dilation of the SB due to MM, an imaging finding usually associated with lymphoma or SB primary tumours, which have different clinical management.

## Case presentations

### Case 1

**Fig. 1:**
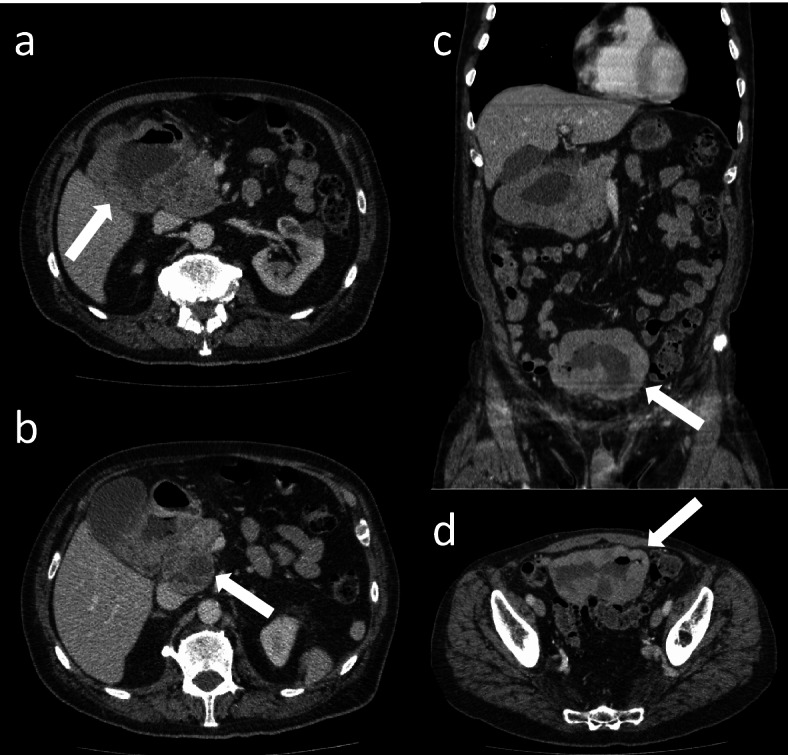
**a** and **b** Axial CT images of the abdomen with a large cavitated aneurysmal mass in the duodenum (arrow in a) with an adjacent necrotic node (arrow in b). **c** coronal image showing the aneurysmal dilatation of the duodenum and ileum (white arrow), the latter also present in **d** in the axial plane

An 80-year-old gentleman presented with a history of weakness, fatigue, breathlessness and chest pain, which he initially attributed to an episode of food poisoning. His past medical history included previous thyroidectomy for a thyroid nodule and radiotherapy-treated prostate cancer. A full blood count demonstrated a mild iron-deficiency anaemia. A CT scan of the chest, abdomen and pelvis was arranged, which identified a large cavitated aneurysmal mass arising from the ileum within the pelvis, adjacent to the sigmoid colon (Fig. 1). A further similar large lesion was identified arising from the first part of the duodenum. Neither of these lesions was causing significant proximal obstruction. Additionally, there was a single heterogeneous 48 mm precaval lymph node as well a right adrenal nodule and several pulmonary nodules. The reporting radiologist felt the findings most likely represented lymphoma.

**Fig. 2:**
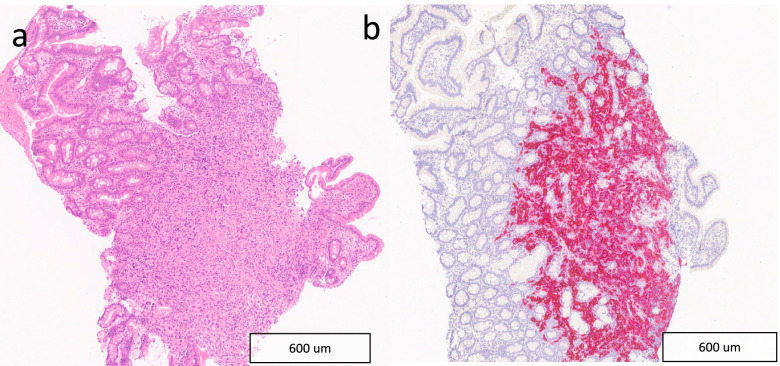
**a** Histological section of native duodenal mucosa infiltrated by atypical cells with irregular nuclear borders and hyperchromasia. **b** The atypical cells showed a melanocytic immunophenotype with expression of Melan A (pictured) and SOX10

The patient underwent oesophago-gastro-duodenoscopy, which found the duodenum to be replaced by tumour. Biopsies of this lesion revealed infiltration of the lamina propria and submucosal by tumour cells. Immunostaining showed the tumour to be compatible with metastatic melanoma with expression of melan-A, SOX10 with negative CKMNF (epithelial marker), CDX2 (Fig. 2); BRAF mutation was not present.

On further questioning, the patient had had a pT4a (Breslow thickness 4.1 mm, mitotic index 1/mm^2^) melanoma excised from his right cheek 5 years previously, for which he declined any further treatment or follow up.

### Case 2

**Fig. 3:**
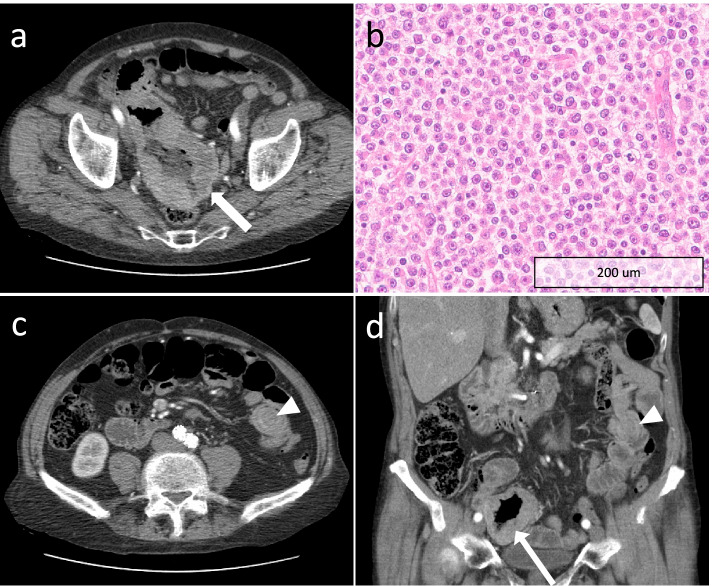
**a** Axial CT of the pelvis with a large necrotic pelvic mass arising from the jejunum with aneurysmal dilatation (white arrow); **b** Histological section of resected jejunum infiltrated by atypical epithelioid cells with prominent nucleoli and conspicuous mitotic activity; **c** axial and **d** coronal reformat CT of the pelvis showing a 2 cm endoluminal deposit (white arrows heads) in the mid jejunum, with an associated degree of intussusception

An 83-year-old man presented with a three-month history of change in bowel habit and a palpable right iliac fossa mass with a positive faecal occult blood test. Whilst being investigated for the new symptoms, a surveillance CT scan was performed for follow up of aortic repair undertaken 2 years before. This scan (Fig. 3) showed an incidental large (12 × 7 cm) necrotic pelvic mass arising from the distal jejunum with aneurysmal dilatation and extension into the right iliac fossa with close contact with the caecum, appendix and distal ileum. There was also a 2 cm endoluminal deposit in the mid jejunum, with associated intussusception. These findings were reported as highly suggestive of small bowel lymphoma and the patient was referred to haematology for review. He subsequently underwent a laparoscopic examination and surgical resection of the distal jejunal mass. Histology and immunohistochemistry confirmed the diagnosis of MM (Fig. 3).

Review of his past medical history revealed a prior diagnosis of pT2a melanoma (Breslow thickness 1.8 mm, mitotic index 22.9/mm^2)^ on his right auricle resected six years before, for which he had been discharged from routine surveillance one year before.

### Case 3

A 65-year-old lady presented with a pT3b melanoma in the left posterior upper arm (Breslow thickness 2.4 mm, mitotic index 8/mm^2^). She underwent wide local excision and sentinel lymph node biopsy of left axilla. Two years later, surveillance imaging identified a solitary lung metastasis, which was treated by video-assisted thoracic surgical lingulectomy. Seven months later, she developed more widespread metastases involving the occipital lobe of the brain and the right adrenal gland. She received stereotactic radiotherapy to the brain lesion and two cycles of ipilimumab and nivolumab immunotherapy. Her immunotherapy was discontinued following grade 3 colitis which required two hospital admissions, intravenous methylprednisolone and infliximab. She remained well and in remission for the next 2 years.

**Fig. 4:**
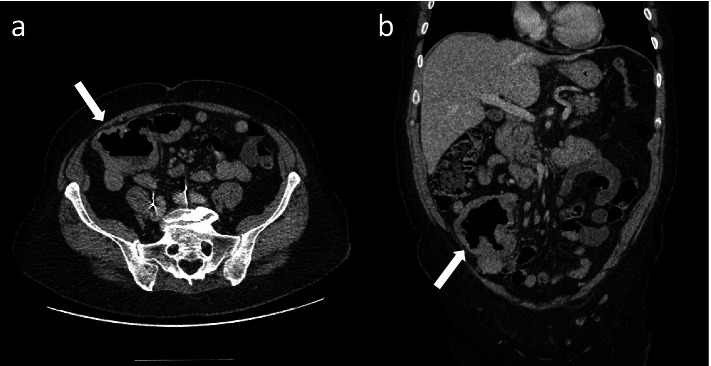
**a** Axial and **b** coronal CT images of the abdomen and pelvis with a large necrotic pelvic mass arising from the mid ileum with aneurysmal dilatation (white arrows)

**Fig. 5:**
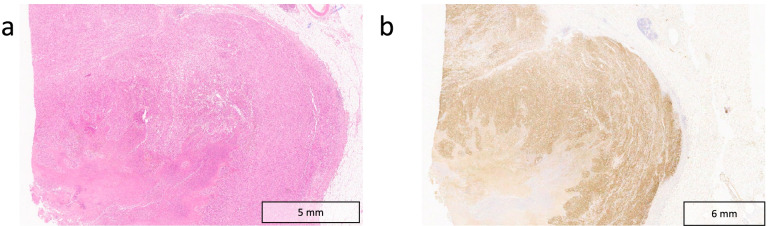
**a** Histological section of small bowel infiltrated by atypical epithelioid cells with prominent nucleoli and abundant melanocytic pigment with background necrosis. **b** Immunohistochemistry shows S100 (pictured), Melan A and HMB45 positivity

However, when symptoms of diarrhoea, abdominal pain and fatigue began 12 h after receiving her flu vaccination, the symptoms were initially considered to be a flare of immune-related colitis. Her symptoms improved transiently with oral steroids and antidiarrhoeal medication. The patient went on to have a flexible sigmoidoscopy, which was limited to the proximal sigmoid due to patient discomfort. The visualised mucosa and obtained mucosal biopsies were normal. Four months later, the patient was admitted to the hospital with worsening of abdominal pain and diarrhoea, where she had an urgent CT scan of the abdomen and pelvis (Fig. 4). The scan demonstrated an 8 cm segment of mid ileum with diffuse thickening and aneurysmatic dilatation in the right iliac fossa, but no upstream bowel dilatation. There was associated mesenteric stranding with a trace of free fluid and lymph nodes measuring up to 10 mm in short axis. The suggested initial imaging diagnosis was small bowel lymphoma. The mass was then surgically resected but the histology confirmed MM (Fig. 5).

Further clinical information of all cases can be found on the supplementary table S1.

## Discussion

Gastrointestinal spread from MM is relatively common, with the SB representing the most common site of involvement [14, 15]. However, SB involvement is still vastly underappreciated clinically, with studies reporting the presence of lesions in 43.5–60% [16, 17] of cases postmortem, but only 1.5–4.4% antemortem [18]. SB melanoma metastases are frequently multiple, due to haematogenous dissemination, and preferentially affect the terminal ileum [19] and jejunum [1]. As in our reported cases, SB metastases are more common with cutaneous as opposed to non-cutaneous melanomas [10]. Primary mucosal melanomas arising in the SB are rare, remaining a controversial diagnosis as the possibility of a MM from an unidentified or regressed primary cutaneous melanoma should always be considered [20, 21].

The clinical presentation of SB MM is usually non-specific, including a constellation of symptoms associated with GI tract pathology, including abdominal pain, unexplained weight loss, iron-deficiency anaemia, change in bowel habit and GI bleeding, though a large proportion may be asymptomatic. Metastases are more frequent in the SB than primary tumours and may occur by haematogenous spread, local extension or intraperitoneal seeding [22]. Melanoma is the most common cancer type to metastasise to the SB [10, 11], occurring either at the time of initial diagnosis, or not uncommonly several years after the primary malignancy has been treated (average of 7.2 years) [11]. In our cohort, we found the SB to be the first site of MM in 2 cases, occurring on average 5 years after resection of the primary melanoma. Other primary tumours that often metastasise to the SB include breast and lung cancer [22].

The diagnosis of SB MM remains challenging due to its vague clinical presentation and limited endoscopic access. Most lesions are identified during surveillance imaging, or incidentally in patients having routine or urgent scans for other reasons. As imaging patterns of SB involvement can be diverse and mimic several other conditions, it is important to keep MM as a possible differential in patients with a prior history of melanoma, even when unusual features, like aneurysmal SB dilatation, are present. In all 3 cases, the initial radiological diagnosis was given as lymphoma, even when secondary imaging clues were present which could have helped raise concern for MM. In one of the cases, the presence of a small number of localised large necrotic lymph nodes close to the main lesion could have prompted the radiologist to consider a different diagnosis besides lymphoma, as necrotic lymph nodes are uncommon in untreated lymphoma [23]. Equally, concomitant SB lesions with diverse appearance and/or the presence of SB intussusception could have raised suspicion for MM. Finally, another tip that can guide the imaging diagnosis towards MM is the identification of other metastatic sites atypical for lymphoma, such as the adrenal glands, subcutaneous tissue and peritoneum.

Interpretation of staging CT studies of patients with MM can be challenging given the need for a thorough review of the scans and unpredictable metastatic patterns with this disease. Nevertheless, the bowel is an important review area in these patients, particularly in the case of primary cutaneous melanoma arising in the head and neck region, trunk and lower extremity [24]. On the other hand, when a SB lesion is incidentally identified on imaging, one should always consider the possibility of MM, and careful medical history should be taken regarding prior history of primary skin lesions.

In the case of a suspected SB lesion and/or in the presence of symptoms, video-capsule is still the preferred method for non-stenotic SB lesions [19]. However, CT/MR enterography/enteroclysis are valuable and more accessible techniques in the detection of small lesions. FDG-PET/CT adds the potential of detection of other secondary lesions, unknown primary or residual tumour [25–28]. Nonetheless, definitive diagnosis of a small bowel metastasis can only be obtained histologically through surgical/endoscopic biopsy.

Modern therapeutic interventions for MM have radically expanded in the last decade and significantly improved overall survival of patients, some able to enter long term remission [29]. Although much of the survival gains are attributable to novel systemic therapies, surgical clearance of oligometastatic disease remains a key life-saving intervention for some. In the case of MM in the GI tract, surgery is key to alleviating symptoms as well as prolonging life. Therefore, early diagnosis and rapid intervention for SB MM is key to maximising quantity and quality of life, even in palliative cases [13].

## Conclusion

Recognition of unusual radiological signs of SB spread from MM, like aneurysmal SB dilatation, is important to expedite diagnosis, provide appropriate treatment, and consequently improve quality of life and likely survival of these patients. As the most common cancer type metastasising to the SB and as a known imaging mimicker, MM should remain in any radiologist’s differential diagnosis for SB lesions. Not uncommonly, there are other imaging features which can help make the correct diagnosis.

## Supplementary Information

**Additional file 1.**

## Data Availability

The datasets used and/or analysed during the current study are available from the corresponding author on reasonable request.

## Abbreviations

CDX2: Caudal-related homeobox gene 2
CD45: Leucocyte common antigen
CT: Computer tomography
GI: Gastrointestinal
HMB45: Human Melanoma Black-45
MM: Malignant melanoma
MR: Magnetic resonance
NGS: Next-generation sequencing
SB: Small bowel
SOX10: Sry-related HMg-Box gene 10
