# Supplementary material for: The forgotten appearance of metastatic melanoma in the small bowel
Source: Cancer Imaging. 2022 Jun 14;22:27. doi: 10.1186/s40644-022-00463-5 (PMC9195247; doi:10.1186/s40644-022-00463-5)
Supplement: Supplementary file 1 — Additional file 1. [file 40644_2022_463_MOESM1_ESM.docx]

**Supplementary Table S1**

| **Case** | **1** | **2** | **3** |
| --- | --- | --- | --- |
| **Ethnicity** | White British | White British | White British |
| **History of long-standing sun-exposure** | Some occupational exposure | Not particular | Not particular |
| **Initial staging**  **(N and M)** | Not documented | N0M0 | N0M0 |
| **Sentinel lymph node localisation** | Declined | Not undertaken | Yes |
| **FDG-PET-scan at initial diagnosis** | No | No | No |
| **Excised margins at diagnosis** | Margins involved – Patient declined WLE and follow up | Margins negative | Margins involved |
| **Systemic treatment** | No | No | Yes, 2 cycles of immunotherapy with ipilimumab and nivolumab |
| **Follow-up** | Declined follow up | Regular follow up for 5 years. Returned to surveillance after SB metastasis. | Regular follow up – still under surveillance |

Supplementary table S1 - Clinical details for each case at the time of diagnosis.
